# Evaluation of *SPARC* as a candidate gene of juvenile-onset primary open-angle glaucoma by mutation and copy number analyses

**Published:** 2010-10-08

**Authors:** Li Jia Chen, Pancy O.S. Tam, Clement C.Y. Tham, Xiao Ying Liang, Sylvia W.Y. Chiang, Oscar Canlas, Robert Ritch, Douglas J. Rhee, Chi Pui Pang

**Affiliations:** 1 Department of Ophthalmology and Visual Sciences, the Chinese University of Hong Kong, Hong Kong, China; 2 Jose B. Lingad Memorial Regional Hospital, San Fernando, Philippines; 3 Department of Ophthalmology, New York Eye and Ear Infirmary, New York, NY; 4 Department of Ophthalmology, Massachusetts Eye & Ear Infirmary, Harvard Medical School, Boston, MA

## Abstract

**Purpose:**

To investigate the involvement of *SPARC* (secreted protein acidic and rich in cysteine) mutations and copy number variation in juvenile-onset primary open-angle glaucoma (JPOAG).

**Methods:**

This study involved the 27 family members from the *GLC1M* (glaucoma 1, open angle, M)-linked Philippine pedigree with JPOAG, 46 unrelated Chinese patients with JPOAG and 95 controls. Mutation screening of the *SPARC* sequence, covering the promoter, 5′-untranslated region (UTR), entire coding regions, exon-intron boundaries, and part of the 3′-UTR, was performed using polymerase chain reaction and direct DNA sequencing. Copy number of the gene was analyzed by three TaqMan copy number assays.

**Results:**

No putative *SPARC* mutation was detected in the Philippine family. In the Chinese participants, 11 sequence variants were detected. Two were novel: IVS2+8G>T and IVS2+32C>T. For the 9 known SNPs, one was synonymous (rs2304052, p.Glu22Glu) and the others were located in noncoding regions. No individual SNP was associated with JPOAG. Five of the most common SNPs, i.e., rs2116780, rs1978707, rs7719521, rs729853, and rs1053411, were contained in a LD (linkage disequilibrium) block. Haplotype-based analysis showed that no haplotype was associated with the disorder. Copy number analysis revealed that all study subjects had two copies of the gene, suggesting no correlation between the copy number of *SPARC* and JPOAG.

**Conclusions:**

We have excluded *SPARC* as the causal gene at the *GLC1M* locus in the Philippine pedigree and, for the first time, revealed that the coding sequences, splice sites and copy number of *SPARC* do not contribute to JPOAG. Further investigations are warranted to unravel the involvement of SPARC in the pathogenesis of other forms of glaucoma.

## Introduction

Glaucoma is a group of degenerative optic neuropathies involving progressive loss of retinal ganglion cells and their axons, resulting in a characteristic pattern of optic nerve head and visual field damage [1,2]. It is the leading cause of irreversible blindness globally [3]. Primary open-angle glaucoma (POAG), characterized by a gonioscopically open anterior chamber angle, is a leading form of glaucoma in many populations [4-6].

POAG has complex etiology. It could be monogenic, e.g., myocilin glaucoma, or multifactorial, resulting from additive or interactive effects of environmental and genetic factors. Intraocular pressure (IOP) is a major risk factor. Accordingly, POAG has been divided into high-tension (HTG, IOP>21 mmHg) and normal-tension (NTG, IOP≤21 mmHg) entities, and it is considered a spectrum of disease reflecting different susceptibilities to a given IOP level [7]. The trabecular meshwork (TM) provides the major resistance to aqueous humor outflow in cases in which IOP is elevated [1,2]. Thus, genetic and/or other factors affecting IOP, outflow facility, and retinal ganglion cell viability may play important roles in POAG susceptibility.

To date, more than 20 linkage loci have been mapped for POAG [8-24]. However, only three genes, i.e., myocilin (*MYOC*, *GLC1A*) [25], optineurin (*OPTN*, *GLC1E*) [26], and WD repeat domain 36 (*WDR36*, *GLC1G*) [18], were identified. These genes account for less than 10% of overall POAG [27-30]. Causal genes in the rest of the loci and genes independent of the linkage loci remain to be identified. Candidate genes for POAG can be prioritized by at least six criteria: (1) expressed in eye tissues; (2) involved in IOP regulation; (3) affecting ganglion cell viability; (4) associated with other eye or retinal diseases; (5) associated with other neurodegenerative diseases; and (6) located at reported linkage loci.

In this study, we evaluated *SPARC* (secreted protein acidic and rich in cysteine, OMIM 182120) as a candidate gene for JPOAG. The *SPARC* gene is located at chromosomal region 5q31.3-q32 within the *GLC1M* locus (5q22.1–32; OMIM 610535) mapped by our group [20]. SPARC, also known as osteonectin or BM-40, is a matricellular glycoprotein that functions primarily to promote extracellular matrix deposition [31]. It is expressed at high levels in bone tissues and is distributed widely in many other tissues and cell types [32]. In human eyes, SPARC is found in lens [33], corneal epithelium [33], TM cells [34,35], and retinal pigment epithelium [33,36,37]. It distributes throughout the trabecular meshwork and is prominent in the juxtacanalicular region [35]. In the trabecular meshwork of postmortem human eyes, *SPARC* and another glaucoma gene *MYOC* responded significantly to elevated-IOP [38]. *SPARC* is one of the most highly upregulated genes in porcine TM cells in response to mechanical stretching [39], supporting an important role of SPARC in IOP regulation [35]. Furthermore, elevated expression of SPARC has been detected in the iris of POAG patients [40], although whether such change was a cause or consequence of glaucoma, or just a phenomenon secondary to the use of topical medications for glaucoma remained unverified. Recently, the SPARC null mouse has been shown to have lower IOP than the wild-type, likely due to decreased outflow resistance. Moreover, heterozygous mice expressed an intermediate phenotype suggestive of a dose-dependent effect of SPARC [41]. These findings suggest that SPARC could be implicated in POAG, likely by compromising the regulation of IOP.

No study has yet evaluated the involvement of *SPARC* mutations in human glaucoma. If any kind of *SPARC* variations are associated with or causative for POAG, at least 5 possibilities should be considered: (1) promoter polymorphisms that affect the expression level of the gene; (2) missense variants with gain (or loss)-of-function; (3) nonsense mutations leading to loss-of-function; (4) variants at the exon-intron boundaries causing alternative splicing; and (5) copy number variants that may alter gene dosage. In view of the finding that SPARC null mice have lower IOPs [41], it is likely additional copies of SPARC may correlate with higher IOP. Moreover, as the *GLC1M* locus was identified in a pedigree of juvenile-onset primary open-angle glaucoma (JPOAG) with high IOP [20], we investigated the involvement of *SPARC* variants in JPOAG by mutation screening and copy number analysis.

## Methods

### Participants

The protocol was approved by the Ethics Committee for Human Research, the Chinese University of Hong Kong. All procedures adhered to the tenets of the Declaration of Helsinki. Written informed consent was obtained from every participant after explanation of the nature of the study.

The Philippine pedigree with JPOAG has been described in detail previously [20,42]. Briefly, this five-generation family comprised 95 members, with 22 being affected. Peripheral venous blood was collected from 27 members, who underwent complete ophthalmic examinations, and of whom 9 were affected. JPOAG was defined based on the following criteria: (1) exclusion of secondary causes, e.g., trauma, uveitis, steroid-induced or exfoliation glaucoma; (2) gonioscopically open anterior chamber angle, Shaffer grade III or IV; (3) IOP≥22 mmHg in the affected eye measured by applanation tonometry; (4) characteristic optic disc damage and/or typical visual field loss by Humphrey automated perimetry using the Glaucoma Hemifield test; and (5) age at diagnosis ≤40 years. For the affected subjects, the age at diagnosis ranged from 12 to 33 years (mean±SD: 19±4.2 years), the highest recorded IOP was between 24 and 44 mmHg (32±6.3 mmHg), vertical cup-disc ratio (VCDR) ranged from 0.7 to 0.9 (median: 0.8), and visual field loss was compatible with glaucoma in two consecutive tests. The unaffected members aged from 3 to 73 years (25±19.9 years) at study recruitment, with IOP <22 mmHg, VCDR between 0.2 and 0.5 (median: 0.3), and the visual field within normal range.

Unrelated Chinese subjects were recruited from the eye clinics of Hong Kong Eye Hospital. We enrolled 46 patients with sporadic JPOAG and 95 controls (characteristics shown in Table 1). They were given complete ophthalmic examinations and were diagnosed using the same criteria described above. Of the patients, age at diagnosis ranged from 6 to 40 years conforming to JPOAG. The highest recorded IOP in the more severely affected eye was between 23 and 69 mmHg and the VCDR 0.5–0.9. Control subjects were recruited from participants aged ≥60 years who visited the clinics for senile cataract, itchy eyes or floaters. They were confirmed to be free of glaucoma or other major eye diseases. Their IOP was <21 mmHg, with both VCDR and visual field within normal range. As SPARC has been implicated in systemic conditions, subjects with known systemic diseases, such as tumor, diabetes, etc., were not included.

**Table 1:** Demographic and clinical characteristics of Chinese JPOAG and control subjects.

			**Age at diagnosis (years)**		IOP (mmHg)*		VCDR*	
**Group**	**Sample size**	**Female (%)**	**Range**	**Mean (SD)**	**Range**	**Median**	**Range**	**Median**
JPOAG	46	19 (41.3)	6–40	24.8 (8.5)	23–69	30	0.5–0.9	0.8
Control	95	40 (42.1)	61–94	75.1 (7.1)	10–21	15	0.2–0.5	0.3

The asterisk indicates that the IOP value is the recorded highest IOP, and the vertical cup/disc ratio (VCDR) is the measure at the latest follow-up visit before study enrollment.

### Gene screening and copy number analysis of *SPARC*

Genomic DNA was extracted from whole blood using the QIAamp DNA Blood Mini Kit (Qiagen, Hilden, Germany) according to the manufacturer’s protocol. DNA concentration was measured by a ND-1000 spectrophotometer (NanoDrop Technologies, Wilmington, DE). The target sequences, including part of the promoter (−1 to −318 bp upstream of the transcription initiation site), 5′-untranslated region (5′-UTR, +1 to +314 bp downstream of the transcription initiation site), the entire coding regions (c.1 to c.912), exon-intron junctions, and part of the 3′-UTR (c.912+1 to c.912+94), were screened in the 27 members of the Philippine pedigree, 46 Chinese patients and 95 Chinese controls. Primer sequences were designed using Primer3 [43] (v.0.4.0) referring to the published gene sequence of *SPARC* (ENSG00000113140) in Ensembl [44] (Table 2**)**. The target sequences were amplified using polymerase chain reaction (PCR) and analyzed by direct DNA sequencing using the dye-termination chemistry (Big-Dye Terminator Cycle Sequencing Reaction Kit; ver. 3.1; Applied Biosystems, Inc. [ABI], Foster City, CA) on an automated sequencer (3130XL; ABI), according to the manufacturer’s protocol.

**Table 2:** Primer sequences and PCR conditions for *SPARC* sequencing.

	**Primer sequence**				
**Amplifying target**	**Forward primer (5′→3′)**	**Reverse primer (5′→3′)**	**MgCl_2_ (mM)**	**Ta (°C)**	**Size (bp)**
SPARC-1 (promoter + exon 1)	CCAGTTCCAAATCATCAAGGA	GGGGTTGGTGCAACTATAGAA	1.5	59	668
SPARC-2 (exon 2)	AAATGGAACCAACCTCCTCA	CAATGGTCCTCATCCCAGTT	1.5	60	388
SPARC-3 (exon 3)	AGCTCCCCTAGCCTGTATCC	CCCTAATTTCTCAGGGCACA	1.5	60	225
SPARC-4 (exon 4)	CTTTCCCTAACACCCCTGGT	TCATGTAGGCTGTCCTCGTG	1.5	60	367
SPARC-5 (exon 5)	TGTGCTAGTCCAGGTGATGC	TGTATTCCGAAGTGCCCAAT	1.5	60	222
SPARC-6 (exon 6)	CAGTGTCCCCATCTCTGAAA	CCCAAGACAGGAGTCTGGAA	1.5	60	250
SPARC-7 (exon 7)	AAGAAACTGTGGCCTGGAGA	CTGGTGCTCAGGGGTAAATG	1.5	60	396
SPARC-8 (exon 8)	CTGGCTAGTCTCTGCCTGCT	TCACTCTAGGGTCTGGGGTCT	2.0	60	279
SPARC-9 (exon 9)	GGGTGTGGAGCTTTTCCAT	CCCCTTGCTTCTTTGTTCAG	1.5	60	229
SPARC-10 (exon 10)	TCCACTGACTCCTTGGGAAG	GGCAGAACAACAAACCATCC	1.5	60	198

The promoter (−1 to −318 bp from the transcription initiation site), 5′-untranslated region (the noncoding exon 1), coding regions, exon-intron boundaries, and a portion of the 3′-untranslated region (+1 to +94 bp downstream the stop codon) were covered by the amplimers. In the table, Ta indicates annealing temperature and Bp indicates base pairs.

Copy number analysis of *SPARC* was performed for all the 27 subjects from the pedigree, 18 randomly selected Chinese JPOAG patients (10 females) and 18 controls (9 females), using the TaqMan^®^ Copy Number Assays (Applied Biosystems). Three assays were selected for this purpose (Table 3), with one being located in proximity to the 5′-end of the *SPARC* gene, one near the 3′-end, and one within the gene. According to the manufacturer’s instructions, the DNA samples were diluted to a concentration of 5 ng/μl. Each PCR reaction mix contained 5.0 μl of 2× TaqMan^®^ Genotyping Master Mix, 0.5 μl of the TaqMan Copy Number target assay, 0.5 μl of the TaqMan Copy Number reference assay (RNase P), which is known to exist only in two copies in a diploid genome, 2.0 μl of Nuclease-free water, and 2.0 μl of DNA. The reactions were processed in an ABI 7900HT Fast real time PCR System using a 384-well reaction plate, with each DNA sample analyzed in duplicates and on 95 °C/10 min for 1 cycle followed by 92 °C/ 15 s and 60 °C/1 min for 40 cycles. Data was collected by the SDS software (version 2.3; ABI) using the standard absolute quantification method. After the reaction, raw data was analyzed using a manual cycle threshold (C_T_) of 0.2 with the automatic baseline on, and then imported to the CopyCaller^TM^ Software (version 1.0; ABI) for post-PCR data analysis. In the software, copy numbers were estimated using a maximum likelihood algorithm. The analytical setting was the same for the three assays.

**Table 3:** TaqMan^®^ Copy Number Assays used for copy number analysis of *SPARC*.

**Assay ID**	**Reporter dye**	**Context sequence**	**Location on NCBI assembly**
Hs02667978_cn	FAM	GTCTCAAAACCCCAGCTCAAAATAC	151021358
Hs06106867_cn	FAM	GTCAGAAGGTTGTTGTCCTCATCCC	151027253
Hs06124887_cn	FAM	CTTCCCAGAGGTGTGGATTAATGGT	151046100

The three assays selected are for target sequences located in proximity to the 5′- and 3′-ends of and within the gene. Any assay(s) detected to have gain or loss of copy number(s) may indicate, at least partially, the copy number of the *SPARC* gene.

### Data analysis

Segregation analysis was conducted for variants detected in the pedigree, including copy number variation. A variant is considered disease-causing if it segregates with disease in the pedigree. For the two novel intronic variants detected in the Chinese subjects, a web-based program Automated Splice Site Analyses (ASSA) [45,46] was used to predict their impacts to alternative splicing. For variants with a minor allele frequency of >1%, Hardy–Weinberg Equilibrium was tested by the χ^2^ test. Variant frequencies between patients and controls were compared by Chi-square test or Fisher’s exact test using SPSS (ver. 16.0; SPSS Inc., Chicago, IL). Linkage disequilibrium (LD) between SNPs and haplotype frequencies were estimated using the E-M algorithm in Haploview [47] and tested for association using χ^2^ analysis. A p<0.05 was considered statistically significant. Since no significant association was detected, correction for multiple testing was not considered.

## Results

### Sequence variants detected in *SPARC*

**Figure 1:**
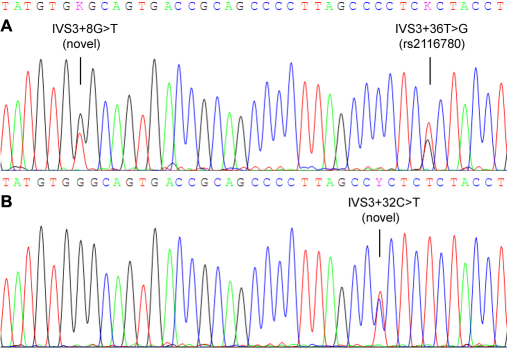
Chromatograms of the novel *SPARC* variants detected in this study. **A**: The variant IVS3+8G>T detected in a control subject, this participant is also heterozygous for the rs2116780:T>G polymorphism. **B**: The variant IVS3+32C>T detected in a Chinese patient with JPOAG.

**Figure 2:**
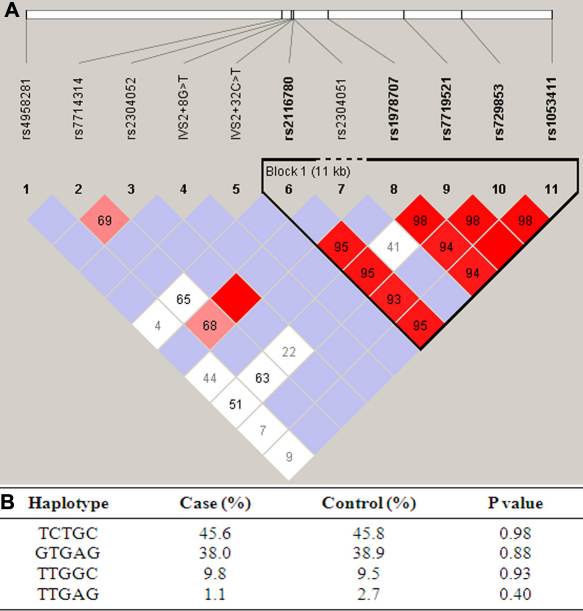
Linkage disequilibrium and haplotype association analyses for the *SPARC* variants detected in this study. **A**: Linkage disequilibrium plot of 11 SNPs of the *SPARC* gene in the combined subjects. D' values corresponding to each SNP pair are expressed as a percentage and shown within the respective square. The five most common SNPs constitute a haplotype block spanning from intron 3 to the 3′-UTR of the gene. **B**: Haplotype-based association analysis of the 5 most common SNPs in the LD block with JPOAG. The frequencies of each haplotype in the patient and control groups were presented in percentage. Only those haplotypes with frequencies >1% were shown.

In the Philippine pedigree, only one *SPARC* variant, namely c.912+29 C>G (rs1053411), was detected in an unaffected subject. No sequence change was found in other family members. In the Chinese study subjects, 11 variants were detected (Table 4), among which two were novel, i.e., IVS2+8G>T and IVS2+32C>T (Figure 1). The heterozygous variant IVS2+8G>T was detected in two (2.1%) control subjects but not in patients, while the IVS2+32C>T was detected in one (2.2%) patient and absent in controls. According to the ASSA program, the two novel variants were predicted to cause no change in the information content of the donor site, suggesting that they are not likely to be functional mutations. The other 9 variants were known polymorphisms in the dbSNP database. Except for a synonymous SNP rs2304052 (p.Glu22Glu) detected in exon 2, all other SNPs were located in noncoding regions. All of these SNPs followed HWE in both the control and patient groups. Moreover, the allele or genotype distribution of each SNP was not significantly different between patients and controls, indicating no association with glaucoma. Linkage disequilibrium analysis revealed an extension of LD throughout the gene. The five most common SNPs, rs2116780 (Intron 3), rs1978707 (Intron 4), rs7719521 (Intron 5), rs729853 (Intron 7), and rs1053411 (3′-untranslated region, 3′-UTR), were contained in a LD block spanning approximately 11 kb (Figure 2A). Haplotype-based association analysis showed that no haplotype was significantly associated with the disorder (Figure 2B).

**Table 4:** *SPARC* variants detected in Chinese JPOAG and control subjects.

				**Minor allele frequency (%)**			**Genotype counts**		
**Location**	**Sequence change**	**Residue change**	**SNP ID**	**Case (n=92)**	**Control (n=190)**	**p**	**Case (n=46)**	**Control (n=95)**	**p**
Exon 1	c.-186G>A*	–	rs4958281	5 (5.4)	6 (3.2)	0.35	0/5/41	0/6/89	0.34
Intron 2	IVS2+56G>C	–	rs7714314	3 (3.3)	7 (3.7)	1.0	0/3/43	1/5/89	0.75
Exon 3	c.66A>G	Glu22Glu	rs2304052	2 (2.2)	5 (2.6)	1.0	0/2/44	0/5/90	1.0
Intron 3	IVS3+8G>T	–	novel	0 (0)	2 (1.1)	-	0/0/46	0/2/93	-
Intron 3	IVS3+32C>T	–	novel	1 (1.1)	0 (0)	-	0/1/45	0/0/95	-
Intron 3	IVS3+36T>G	–	rs2116780	36 (39.1)	77 (40.5)	0.82	7/22/17	15/47/33	0.97
Intron 3	IVS3+42T>C	–	rs2304051	4 (4.3)	6 (3.2)	0.73	0/4/42	0/6/89	0.73
Intron 4	IVS4+31C>T	–	rs1978707	45 (48.9)	92 (48.4)	0.94	10/25/11	24/44/27	0.67
Intron 5	IVS5–59T>G	–	rs7719521	44 (47.8)	89 (46.8)	0.88	10/24/12	22/45/28	0.86
Intron 7	IVS7+100G>A	–	rs729853	38 (41.3)	80 (42.1)	0.90	7/24/15	16/48/31	0.97
3′-UTR	c.912+29C>G	–	rs1053411	39 (42.4)	80 (42.1)	0.96	8/23/15	16/48/31	0.99

*This SNP is located 186 bp upstream the start codon “ATG”; 3′-UTR: 3′-untranslated region.

### Copy number analysis of *SPARC*

**Figure 3:**
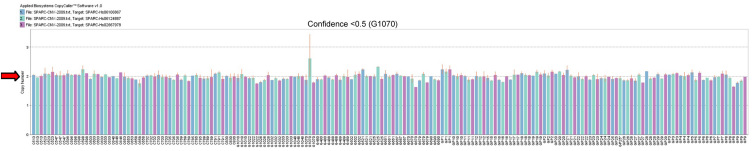
Copy number of the *SPARC* gene in the family members from the Philippine pedigree and the randomly selected Chinese JPOAG patients and controls. Each bar represents the copy number prediction of the target sequence in each subject. And each color presents each copy number assay. Thus, each individual is represented by three bars. The red arrow indicates the reference line for two copies. One sample (G1070) was predicted to have a copy number of 3 but with a confidence of <50%.

The three assays were unequivocally genotyped in all subjects (n=63), with call rates of 100%, to obtain predicted copy numbers of the target sequences in *SPARC* in each subject (Figure 3). The confidence of prediction was greater than 95% for each assay in each subject, except for the sample G1070, with a confidence <50% for assay Hs06124887_cn. This sample was predicted to have 3 copies of the target sequence by this assay but was predicted to have 2 copies of the target sequences by the other two assays with confidence >99%. Therefore, this subject is more likely to have 2 gene copies. All the other samples were predicted to have 2 copies of the gene by any assay, suggesting no correlation between the copy number of *SPARC* and JPOAG.

## Discussion

JPOAG is a subset of POAG characterized by an early age of onset and severe elevations of intraocular pressure. It is often inherited in an autosomal dominant pattern [20], but sporadic cases exist. Thick, compact tissue and extracellular deposits have been found in trabecular meshwork specimens obtained from JPOAG patients during trabeculectomy [48]. It is thus likely that the abnormal trabecular meshwork and aqueous outflow could result in an elevation of IOP and subsequently the glaucomatous changes. The identification of the myocilin gene accounting mainly for JPOAG [25] and its mutant products leading to elevated IOP [2] also supports the possibility that other genes affecting the structure of trabecular meshwork and outflow resistance may function to regulate IOP and, once mutated, increase the risk of glaucoma. However, identification of such genes has since been unfruitful. The tendency for familial inheritance in JPOAG has facilitated the mapping of linkage loci for this phenotype, and at least five loci have been identified, including *GLC1A* (1q21–31, *MYOC*) [8,25], *GLC1J* (9q22) [16], *GLC1K* (20p12) [16], *GLC1M* (5q22.1–32) [20], and *GLC1N* (15q22–24) [21]. Thus, except for *GLC1A*, investigating genes on the other loci may lead to the discovery of new disease genes for JPOAG.

We have previously mapped the *GLC1M* locus in a Philippine pedigree with JPOAG, in which affected family members had moderate IOP elevation (mean±SD: 32±6.3 mmHg) [20]. Therefore, it is likely that this locus harbors a gene involved in IOP regulation. We selected *SPARC* as a candidate causative gene for glaucoma mainly based on that the SPARC protein may function to promote extracellular matrix deposition [31] and is rich in eye tissues, especially in the trabecular meshwork and the juxtacanalicular region [35], that *SPARC* in TM cells is regulated by elevated IOP and mechanical stretching [38,39], that SPARC null mice have lower IOP [41], and that *SPARC* has been mapped to 5q31.3-q32 within the *GLC1M* (5q22.1–32) locus. However, we did not find any putative mutation or copy number variants in the affected and unaffected subjects from the *GLC1M*-linked Philippine pedigree. Although some members, e.g., the 3-year-old unaffected subject, may develop glaucoma later in life, it is likely to be independent of SPARC. As such, *SPARC* could be excluded as the gene responsible for the linkage signal. Discrepancy is known to exist between genetic and physical maps. According to the Ensembl database [44], *SPARC* (ENSG00000113140) is physically located in the region of 151,040,657–151,066,726 bp at chromosome 5. This region is outside the critical interval defined by the makers D5S2051 (111,009,257–111,009,520) and D5S2090 (147,230,043–147,230,236) [20]. Therefore, although *SPARC* by itself is a good candidate gene for glaucoma, it may not be responsible for JPOAG in the pedigree. As such, the causal gene for glaucoma at the *GLC1M* locus remains to be identified. Recently, with the advent of the next-generation sequencing platform [49], the sequencing capacity has been greatly enhanced. It has been used successfully in pinpointing the genes for some Mendelian disorders [50,51]. Such technologies should speed up the identification of glaucoma genes.

We did not detect any missense changes in *SPARC* in a group of unrelated JPOAG patients and controls. Moreover, of the 11 variants detected, none was associated with glaucoma, either individually or involved in a haplotype. Our sample of 46 patients might be small to provide adequate statistical power to detect the significance. However, the distributions of the genotypes were drastically similar between the patients and controls. It is unlikely that the lack of association was due to insufficient power. Therefore, we expect that *SPARC* gene variants do not have a major contribution to JPOAG genetics. However, it is still possible that rare variants in this gene may contribute to a small portion of patients, which awaits confirmation by screening the gene in a very large sample. It is also possible that variants located outside the coding region of the gene, e.g., those at the 3′-UTR, may contribute to the disease. It has been reported that a SNP at the 3′-UTR of *SPARC*, i.e., +998C>G (equivalent to SNP rs1053411 in the present study), was associated with systemic sclerosis in different populations, and the C/C genotype was correlated with a longer mRNA half-life in normal fibroblasts, than were heterozygotes (G/C). And it was suggested that this may contribute, at least in part, to increased *SPARC* gene expression [52]. However, such association could not be reproduced in another cohort of Caucasian patients [53]. In our study, no significant association was detected for rs1053411, which is in strong LD with the other common SNPs detected. According to the international HapMap project, the SNPs that located at the 3′-UTR of *SPARC* are also in strong LD (data not shown). Therefore, other common SNPs at the 3′-UTR are not likely to be associated with JPOAG, because the haplotypes detected in this study did not have disease association (Figure 2B). As such, if the correlation between C/C genotype at rs1053411 and an increased-SPARC expression is real, as that suggested by Zhou et al. [52], it is likely that such an extent of increased-SPARC expression is not causative for glaucoma. However, whether a more severely elevated expression of the protein, if triggered by other pathological factors, can cause glaucoma remains to be further investigated.

So far, the involvement of the SPARC protein level in the pathogenesis of glaucoma is unknown. SPARC occurs widely in extracellular matrices and is predominantly expressed during embryogenesis and in adult tissues undergoing remodeling or repair. It is believed to play a modulatory role in cell-cell and cell-matrix interactions, differentiation, ECM production and organization, wound healing, and angiogenesis [54-56], suggesting a fundamental role of SPARC to living cells [56]. SPARC has been implicated in multiple systemic as well as ocular conditions. For example, it is expressed at high levels in bone tissues and acts as a major non-collagenous protein of the bone matrix [32,57]. SPARC null mice have low-turnover osteopenia [58], and it was suggested that SPARC may strengthen bone [59]. Moreover, *SPARC* 3′-UTR polymorphisms had been associated with bone density in Caucasian men with idiopathic osteoporosis [60]. Likewise, SPARC null mice have lower IOP [41]. It could thus be hypothesized that increased-SPARC level may elevate IOP and predispose to glaucoma. In the eye, increased SPARC level has been correlated with cataract [61], corneal wound repair [62], and proliferative diabetic retinopathy [63]. In glaucoma, elevated SPARC expression has been detected in the iris of POAG and primary angle closure glaucoma patients [40]. All these findings, in addition to the genetic findings of this present study, suggest that certain SPARC expression level could play a role in eye diseases. However, the correlation between the expression of SPARC and the occurrence of these eye diseases remained to be clarified. Recently, it has been found that SPARC deficiency in mice resulted in improved surgical survival in a mouse model of glaucoma filtration surgery [64]. Whether the SPARC levels in glaucoma patients is correlated with the success rate of filtration surgery is unknown. If such correlation exists, a pre-operative detection of the SPARC level may help with a better treatment plan.

This study is one of several attempts to evaluate the involvement of copy number variation in POAG. Abu-Amero et al. [65] screened 27 Caucasian and African-American POAG patients and 12 ethnically matched controls for chromosomal copy number alterations using high resolution array comparative genomic hybridization. No chromosomal deletions or duplications were detected in POAG patients compared to controls [65]. Davis et al. [66] performed a whole-genome copy number screening in a cohort of 400 patients with POAG and 100 controls and found that rare copy number variations in the *DMXL1*, *TULP3*, and *PAK7* genes may affect development of POAG. Interestingly, the *DMXL1* gene at 5q23.1 is located within the *GLC1M* locus. This suggests that copy number of certain genes at this locus, including *DMXL1*, may contribute to the genetics of POAG. However, our findings in that all of the 27 subjects from the Philippine family and the 36 Chinese subjects were detected to carry two copies of the *SPARC* gene, indicate that copy number variation of *SPARC* is at least not a common phenomenon in the two populations and thus unlikely to be a major genetic contributor to JPOAG. Whether copy number variations in other genes at the *GLC1M* locus, such as *DMXL1*, contribute to glaucoma remain to be investigated.

In summary, by mutation screening and copy number analysis, we have excluded *SPARC* as the causal gene at the *GLC1M* locus in the Philippine pedigree with JPOAG. Our results also suggest that *SPARC* is unlikely to be a major disease causative or associated gene of JPOAG. Further investigations are warranted to unravel the involvement of SPARC in the pathogenesis of glaucoma, and also to identify the causal gene at *GLC1M* for JPOAG.
